# Induction of Fas Mediated Caspase-8 Independent Apoptosis in Immune Cells by *Armigeres subalbatus* Saliva

**DOI:** 10.1371/journal.pone.0041145

**Published:** 2012-07-17

**Authors:** Shanshan Liu, David J. Kelvin, Alberto J. Leon, Liqun Jin, Amber Farooqui

**Affiliations:** 1 Division of Immunology, International Institute of Infection and Immunity, Shantou University Medical College, Shantou, People’s Republic of China; 2 Department of Pathogen Biology, Shantou University Medical College, Shantou, People’s Republic of China; 3 Division of Experimental Therapeutics, Toronto General Research Institute, University Health Network, Toronto, Ontario, Canada; 4 Department of Biomedical Sciences, University of Sassari, Sassari, Italy; University of Sassari, Italy

## Abstract

**Background:**

It is widely recognized that the introduction of saliva of bloodsucking arthropods at the site of pathogen transmission might play a central role in vector-borne infections. However, how the interaction between salivary components and the host immune system takes place and which physiological processes this leads to has yet to be investigated. *Armigeres subalbatus* is one of the prominent types of mosquitoes involved in the transmission of parasitic and viral diseases in humans and animals.

**Methodology/Principal Findings:**

Using murine peritoneal macrophages and lymphocytes, and human peripheral mononuclear cells (PBMCs), this study shows that saliva of the female *Ar. subalbatus* induces apoptosis via interaction with the Fas receptor within a few hours but without activating caspase-8. The process further activates downstream p38 MAPK signaling, a cascade that leads to the induction of apoptosis in capase-3 dependent manner. We further illustrate that *Ar. subalbatus* saliva suppresses proinflammatory cytokines without changing IL-10 levels, which might happen as a result of apoptosis.

**Conclusions:**

Our study shows for the first time that saliva-induced apoptosis is the leading phenomenon exerted by *Ar.* *subalbatus* that impede immune cells leading to the suppression of their effecter mechanism.

## Introduction

Vector borne diseases constituting a number of viral, bacterial and parasitic infections cause significant morbidity and mortality in humans and animals. The diseases were considered to be limited to tropical and subtropical geographical regions due to their favorable climatic conditions for vector breeding; however, their global spread in recent years indicates that the ability of arthropod vectors to breed and transmit diseases is not restricted to climatic boundaries [1]. Arthropod vectors have species specificities in the transmission of infection due to their blood-sucking preferences. Anthropophilic mosquitoes, such as *Anopheles gambiae*, prefer to bite humans and transmit malarial parasites, while *Aedes albopictus* and *Ae. aegypti* transmit arboviruses such as dengue, chikungunya, yellow fever virus. On the other hand, some mosquito species also carry zoophilic characteristics so they favor of other animals’ blood and transmit heartworm, malaria and arborviruses to livestock and canines [2].

*Ar. Subalbatus*, one of the prominent mosquito vectors carrying both anthropophilic and zoophilic characteristics, is commonly found in rural areas during twilight hours. For animals, it acts as an intermediate vector for the transmission of several parasitic diseases such as dirofilariasis (heartworm disease) caused by *Dirofilaria immitis* [3]. The disease causes significant damage to live farming around the globe, especially in tropical and temperate regions in the United States, Europe, Australia, South America and Japan [4]. Another parasitic disease transmitted by *Ar. subalbatus* is lymphatic filariasis (LF). It is widely accepted that *Ar. subalbatus* carries filarial nematodes such as *Brugia timori, B. malayi* and *B. pahangi* and transmits them to humans and canines upon biting [5]. Although the mortality rate of LF is not high, it causes permanent damage to limbs and genital organs and may causes elephantiasis and hydrocele. It is estimated that 120 million people have LF globally and approximately 1.1 billion are at the risk of becoming infected [6].

*Ar. subalbatus* also act as natural vector for several viral infections such as Japanese encephalitis (JE) and West Nile Fever, widespread mosquito-borne flaviviruses that cause viral encephalitis and are responsible of high mortality and morbidity worldwide. JE, which is endemic in several Asia Pacific regions, causes an estimated 35,000 to 50,000 infections and 10,000 to 15,000 deaths annually, leavings around 50% of survivors with lingering neurological effects [7]. A recent report also suggests that a novel dsRNA virus, “totivirus”, which is carried by *Ar. Subalbatus*, can be replicated in mammalian systems, and might also be involved in the transmission of other viral diseases [8].

During mosquito-borne disease transmission, it is widely accepted that salivary contents may be responsible for the transmission of certain pathogens but they are refractory to the others [5]. However, less is known about the interaction of saliva with disease processes. Accumulated evidence suggests that mosquito saliva might engage the host immune system that indirectly favours disease establishment [9], [10]. Salivary gland extract (SGE) of the female *Ae. aegypti* has been found to suppress iNOS, IFN-β mRNA expression in antigen presenting cells (APCs) in the absence of virus [11]. Another study proved that components of SGE of *Ae. aegypti* (L.) and *Culex quinquefasciatus* (*Cx. quinquefasciatus*) can neutralize the effector function of immune cells by blocking cell proliferation and secretion of Th1 and Th2 cytokines [12], [13]. However, less is known about the interactions between the vector salivary gland components and the host immune response; detailed studies elucidating the molecular mechanism(s) are still needed.

Southern China is located in the subtropics. Due to warm, humid weather and compromised environmental hygiene, the region acts as a natural reservoir for mosquito breeding with a high incidence rate of mosquito-borne infections such as dengue, JEV, malaria and lymphatic filariasis [14], [15], [16]. In 2005, it was reported that *Ar. subalbatus* was involved in the transmission of 53 human cases of JE in Jieyang city located in Guangdong, the southern province of China [17]. Moreover, *Ar. subalbatus* is an integral part of the mosquito fauna of the region and may contribute in disease spread [18]. These observations stimulated our interest to study the interaction of *Ar. subalbatus* saliva with immune cells to understand mosquito saliva’s potential role in disease processes. This study provides novel insights about cellular engagement and the molecular mechanism of the *Ar. subalbatus* salivary gland with different immune cells using mononuclear phagocytes and lymphocytes as models due to their ability to recognize and present antigen and activate cell mediated immune responses respectively.

## Methods

### Ethics Statement

Ethical approval for the study including the use of animals and human subjects was obtained from ethical committee of Shantou University Medical College, Shantou, GD, China. Animal experiments were performed in accordance with standard protocols approved by institutional Animal Care and Use Committee. For the collection of peripheral blood, healthy donors were recruited on their own will. Written consents were obtained from each participant.

### Material

Dulbecco’s Modified Eagle medium (DMEM) and RPMI 1640 cell culture media and other essential reagents were obtained from Invitrogen (Shanghai, China). Anti caspase-3(p8) antibody was purchased from Santa Cruz Biotechnology (CA, USA). The following antibodies, CD11b-APC, CD11c-efluor 450, Ly6G/c-PE, and CD3-Alexa fluor 647, were purchased from ebiosciences Inc. (CA, USA). Cy-3-conjugated donkey anti-goat antibody from Beyotime (Shanghai, China) and Anti-Fas and capase-8 antibodies from Biosynthesis Biotechnology Co (Beijing, China) were used. Recombinant mouse interferon gamma (IFN-γ) was obtained from R & D Systems (Minneapolis, MN, USA). SB202190 (p38 inhibitor) and SP600125 (JNK inhibitor) were supplied from Sigma (St Louis, MO, USA). Annexin-V-FITC apoptosis detection Kit and caspase-3 colorimetric assay kit were purchased from KeyGEN BioTECH (Nanjing, China). Human acute monocytic leukemia cells (THP-1, ATCC) were kindly donated by Prof. Jiang Jikai, Biochemistry Laboratory of Shantou University Medical College. Female, 6- to 12-week-old BALB/C mice were obtained from Vital River Laboratory (Beijing, China). They were maintained on a standard animal diet in a specific pathogen free (SPF) facility of University of Shantou Medical College with controlled temperature and humidity.

### Mosquito Feeding and Collection of Salivary Glands

The larvae and pupae of *Ar. subalbatus* were field-collected from Mianhu village of Jieyang county, Guangdong province, China. Mosquito species were identified mainly by the fourth instar larvae and by both male and female adult stages according to the keys from Belkin and Bolin Lu [19]. The larvae and pupae were maintained in wide-mouth bottles containing half tap water and half dirty water collected from the breeding sites in mosquito cages at 26°C with relative humidity of 70% for 2–3 days for pupae and 5–8 days for larvae to complete the developmental stages for emergence. Adult mosquitoes were then fed on 10% sucrose solution for 5–10 days and used for dissecting salivary glands.

To collect salivary glands, mosquitoes were anesthetized by ether, chilled on ice and disinfected with 75% ethanol. Salivary glands from male and female mosquitoes were dissected under a microscope in sterile PBS containing 5% glycerol and stored at −80°C in aliquots until used. For the preparation of SGE, salivary glands were kept on ice and sonicated (JY92-II, SCIENTZ, Ninabo, China) at 40% duty cycle at 400 W for 8 seconds followed by centrifugation at 14,000×g for 10 minutes at 4°C. Supernatant containing clear salivary gland proteins were collected and the concentration was determined by the Bradford protein assay kit (Bradford). To ensure the uniformity in the composition and extraction procedure, salivary gland extract was prepared in stock and divided into 50 µl aliquots which were stored at −80°C. Each tube was used only once to avoid protein degradation due to freeze-thaw cycles.

### Isolation and Culture of Primary Cells and Cell Lines

Macrophages were collected from the unstimulated peritoneal cavity of BALB/C mice and resuspended in DMEM supplemented with 10% fetal bovine serum, 100 U/ml penicillin, 100 µg/ml streptomycin, 200 U/ml gentamicin and 1 µg/ml amphotericin B (hereafter termed as DMEM). Cells were seeded in 6-well plates at the density of 10^6^. Following incubation for 2 hours, non-adherent cells were washed off and the adherent macrophages were cultured in DMEM. For flow cytometry experiments, peritoneal cells were added to 1.5 ml sterile eppendorf tubes with the final density of 1×10^6^ cells followed by treatment with salivary gland proteins or vehicle and incubation at 37°C in 5% CO_2_ for indicated time period. Selective gating on the cells with phenotype Ly6G/c^−^ CD11b^+^ CD11c^−^ was applied to identify macrophages.

For the collection of splenocytes, spleens of BALB/C mice were collected, homogenized in DMEM and filtered through 40 µm filter (BD) to get a single-cell suspension. Subsequently, cell suspensions were centrifuged at 400×g for 10 minutes at 4°C and the pellets were incubated with 2 ml erythrocyte lysis buffer (Solarbio) on ice for 3 min. Following the lysis of erythrocytes, splenocytes were washed twice with DMEM and cultured in 24-well cell culture plates (Nunc) at the density of 1×10^6^ cells. For flow cytometry, only CD3^+^cells were considered as lymphocytes.

Human peripheral blood mononuclear cells (PBMCs) were isolated from peripheral blood samples obtained from healthy donors using Ficoll density gradient centrifugation. Cells were cultured in DMEM. For flow cytometry, CD3^+^ and CD14^+^ cells were considered as lymphocytes and monocytes respectively.

Murine leukemic monocytes macrophages (Raw 264.7) cells obtained from ATCC were cultured in DMEM supplemented with 10% fetal bovine serum and antibiotics. THP-1 cells were cultured in RPMI 1640 supplemented with 10% fetal bovine serum, 0.05 mM 2-mercaptoethanol, and antibiotics.

Cells were treated with indicated concentrations of SGE for 24 hours at 37°C in a humidified 5% CO_2_ incubator. Similar treatment with vehicle (5% glycerol/PBS) was given to control groups. For certain experiments, cells were further stimulated with IFN-γ for 48 hours.

### Real Time RT-PCR Analysis

Cellular RNA were purified using Trizol (Invitrogen), reverse transcribed with the high-capacity cDNA RT kit (Applied Biosystems, Foster City, USA) and then amplified using SYBR Green master mix (Invitrogen) with 0.5 pmol/µl of forward and reverse primers of each specific gene. The β-actin, TNF-α, CXCL10, IFN-β, STAT1, iNOS were detected. The following primers were used: β-actin (forward primer AGGGAAATTGTTCGTGACATAAA, reverse primer TCATAACTCTTCTCCAAGGAGG), TNF-α (forward primer CATCTTCTCAAAATTCGAGTGACAA, reverse primer TGGGAGTAGACAAGGTACAACCC), CXCL10 (forward primer GGTTGC CAAGCCTTATCGGA, reverse primer ACCTGCTCCACTGCCTTGCT),

IFN-β (forward primer TGGAAAGATCAACCTCACCTAC, reverse primer CATTCTGGAGCATCTCTTGG), STAT1 (forward primer CGCTGGGAACAGAACTAAT, reverse primer CAAAGACCTCCAGGTCAATC), iNOs (forward primer CGCAAGAGAGTGCTGTT, reverse primer TGGTAGCCACATCCCGA), IL-10 (forward primer TACAGCCGGGAAGACAAT, reverse primer TACAGCCGGGAAGACAAT). Relative gene expression was calculated after normalization with β*-actin* gene. Amplification conditions will be provided upon request.

### Cell Viability Assay

To detect whether SGE have a cytotoxic effect on cells, peritoneal macrophages were incubated in the presence or absence of SGE for 48 h at a density of 5×10^4^ cells in each well of 96-well plate. Cells were added with 3-(4,5-dimethylthiazol-2-yl)-2,5-diphe-nyltetrazolium bromide (5 mg/ml, SIGMA) for 4 h at 37°C in the dark followed by the addition of 200 µl dimethylsulphoxide (Generay Biotech Co.Ltd.). Absorbance was measured at 570 nm using a Titertek Multiskan automatic microplate reader.

### Measurement of Nitrite Production

Nitric oxide levels were measured by detecting the nitrite in cell supernatants of SGE or vehicle treated cells using Griess reagents according to the manufacturer’s instructions. Briefly, equal volumes of cell supernatant and Griess reagent were mixed in each well of a 96-well plate and incubated for 5 minutes. The absorbance was read at 540 nm. The nitrite production of each sample was quantified with reference to a standard curve constructed with serially diluted sodium nitrite.

### Flow Cytometry

The Annexin-V FITC apoptosis detection kit was used to evaluate the cell apoptosis. Briefly, following 24 hours of treatment with SGE or vehicle, cells were washed twice with cold phosphate-buffered saline (PBS) and stained with 5 µl of AnnexinV-FITC and 5 µl of propidium iodide (PI) per sample. The cells were incubated at room temperature for 10 minutes. For surface expression of Fas/CD95, peritoneal macrophages were incubated in the presence or absence of SGE for 24 hours, washed twice with PBS and incubated with purified CD16/CD32 antibody for 5 minutes at room temperature to prevent nonspecific binding followed by incubation with 2.5 µg of Fas/CD95 antibody for 30 minutes at room temperature. Fluorescence labeling was performed using dylight649-conjugated donkey anti-rabbit secondary antibody with the concentration of 1∶500 for 30 minutes in the dark at room temperature. Cells were then washed and resuspended in PBS with 10% FBS. A similar procedure was followed for the detection of intracellular caspase-8.

For the identification of cell type, cells were stained with surface markers per the above mentioned protocol. Macrophages were identified by the presence of surface phenotype of CD11b+ Ly6G/c- CD11c- whereas lymphocytes were identified as CD3+. Data was acquired on a BD FACSAria II flow cytometer (BD Biosciences) and Flowjo software (Treestar, Inc. SanCarlos, CA) was used for data analysis.

### Detection of Caspase3

To detect whether caspase3 was activated during apoptosis triggered by SGE, cells were cultured in a 24-well format on sterile cover slips and stimulated in the presence or absence of SGE for 24 hours. They were washed three times with HBSS and fixed with 4% paraformaldehyde for 30 minutes. Permeabilization was achieved using 0.5% Triton X-100 for 30 minutes at room temperature followed by blocking with 5% BSA for 1 hour at room temperature and overnight incubation with anti-caspase-3p20 at 4°C. Caspase-3p20 was stained using cy-3-conjugated secondary anti-goat antibody for 1 hour at room temperature. 4′,6-diamidino-2-phenylindole (DAPI) was used to stain the nucleus. Cellular expression of Caspase-3p20 was captured by fluorescence microscope (Nikon, Japan).

### Statistical Analysis

Data was analyzed by PAWS Statistics 18 (SPSS Inc., Chicago, IL, USA). The values were expressed as mean ± SD in the figures. Two-tailed student-t test and One way ANOVA were applied to assess statistical significance of the differences between two and more than two groups respectively. In case of One way ANOVA, Tukey’s post test analysis was performed to see statistical differences between the groups. A p value less than 0.05 was considered significant.

## Results

### *Ar. subalbatus* Saliva Inhibit Physiological Mediators of Immune Response

**Figure 1:**
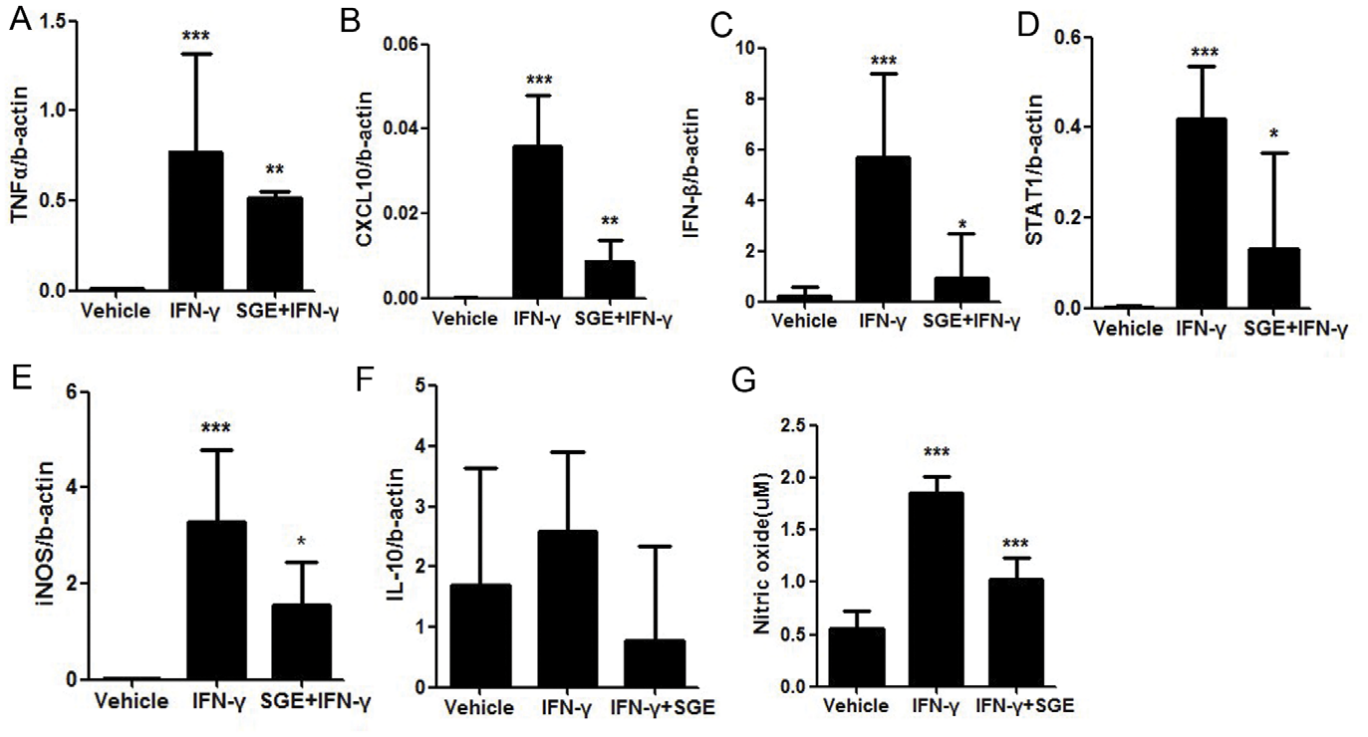
The effect of SGE on physiological mediators of immune response in peritoneal macrophages. Peritoneal macrophages were pretreated with female SGE (20 ug/ml) or vehicle for 3 h followed by the addition of IFN-γ (50 U/ml). Cells were incubated for 48 h and gene expression was determined by real time PCR. Data was normalized with *β*-actin mRNA expression (A–F). Nitric oxide production in culture medium was measured by Griess test (G).The data was showed as means ± SD (F). (* P<0.05; ** P<0.01).

Cytokines and chemokines are produced by host cells, upon pathogen entry. Therefore, we assessed whether *Ar. subalbatus* SGE affects initial mediators of the host immune response. Peritoneal macrophages were initially treated with 20 µg/ml of SGE from the female *Ar. subalbatus* for 3 hours and subsequently stimulated with 50 U/ml of IFN-γ, a known inducer of inflammatory response. First we confirmed that IFN-γ was able to induce cytokine/chemokine expression in peritoneal macrophages. Gene expression analysis further revealed that SGE significantly downregulates IFN-γ induced mRNA expression of CXCL10 (P<0.01) and TNF-α (P<0.01) in macrophages (Figure 1). We also observed that SGE was able to mute integral components of macrophages’ antiviral response such as IFN-β (P<0.01) and cytoplasmic transcription factor, STAT1 (P<0.01); however, no significant difference in the expression of these molecules was observed after treatment with 10 µg/ml of SGE (data not shown). Since STAT1 expression is connected to nitric oxide (NO) generation, which is another important physiological mediator during antiviral and antiparasitic responses, we examined the effect of SGE on NO production at transcriptional and post translational levels. mRNA profile of SGE treated macrophages showed reduction inducible nitric oxide synthase (iNOS) expression (P<0.01) which was further confirmed by measuring NO levels using the Griess test (Fig.1 G ). The results showed that 20 µg/ml of SGE can prevent NO production in IFN-γ induced macrophages compared to those cells which did not receive the SGE treatment (P<0.0001). Moreover, significant reduction in NO production was observed in macrophages treated with 10 µg/ml of SGE (Data not shown). Taken together, the results indicate that SGE negatively regulates the host defense mechanism in a manner independent of pathogen-associated factors and amount of saliva. There could be two possibilities accounting for these results. First, female SGE induces anti inflammatory cytokines so it may directly suppress physiological immune mediators or it may trigger apoptosis in peritoneal macrophages, which may eventually lead to the down regulation of immune mediators. We found that SGE did not alter mRNA expression of IL-10 which is an anti-inflammatory cytokine secreted by monocytes and macrophages and capable of inhibiting pro-inflammatory cytokines.

### *Ar. subalbatus* Saliva Induce Apoptosis in Peritoneal Macrophages

**Figure 2:**
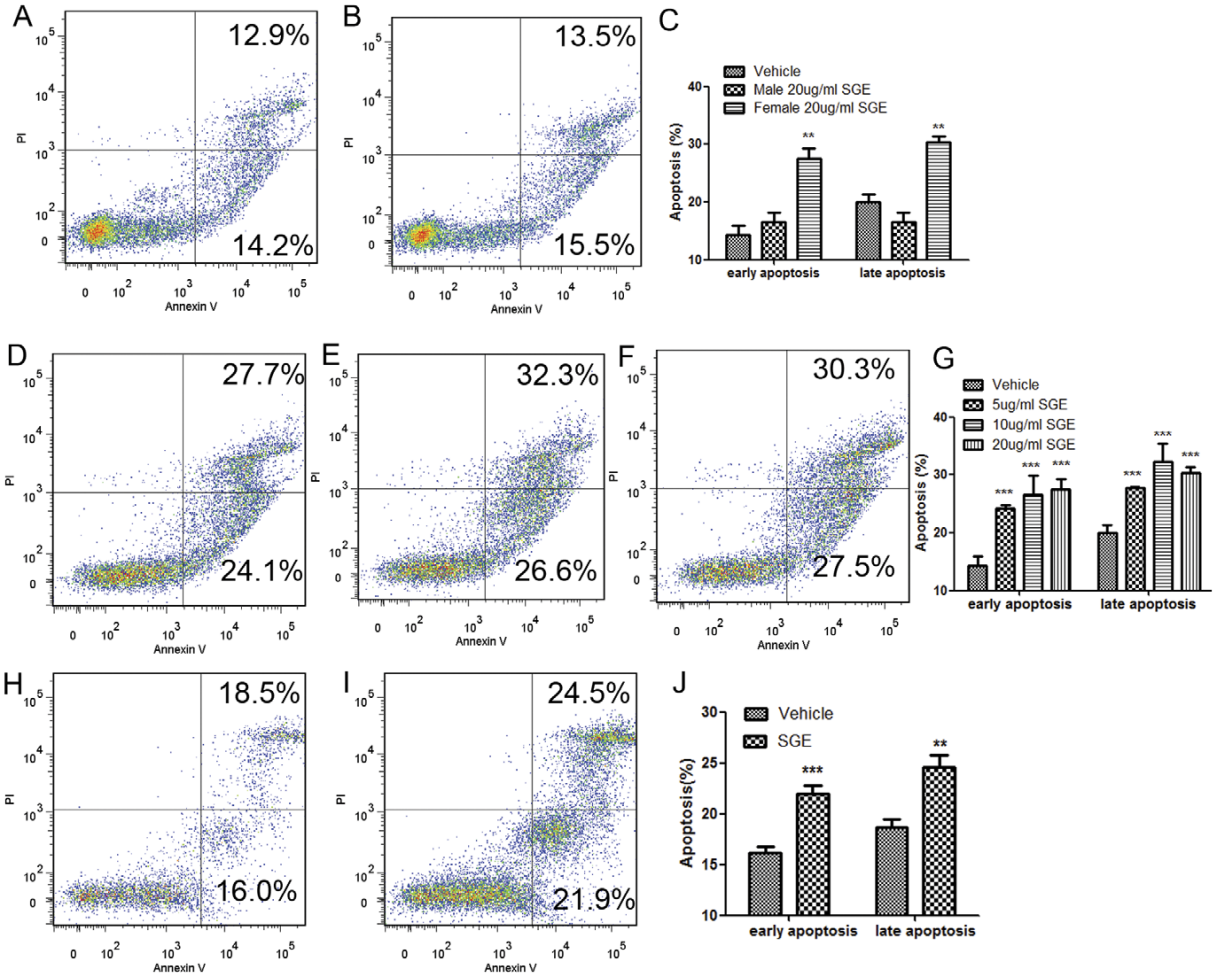
SGE triggers apoptosis in peritoneal macrophages. FACS plots show the pattern of apoptosis in representative samples from each group. For the comparison between male and female SGE-induced apoptosis, peritoneal macrophages were incubated in the presence of (A) vehicle, (B) male SGE (20 µg/ml) and (F) female SGE (20 µg/ml) for 24 hours. To determine concentration dependent effect, cells were also incubated with (D) 5 µg/ml and (E) 10 µg/ml of female SGE. For time dependent studies, cells were incubated with (H) vehicle and (I) 20 µg/ml of female SGE for 6 hrs. Apoptosis was analyzed by Annexin-V FITC and PI staining by flow cytometry. AnnexinV-, PI- cells are live cells, annexinV+, PI- cells are early apoptotic cells, and annexin V+, PI+ cells are late apoptotic cells. Comparisons in the form of mean ± SD and statistical significance are presented between (C) male and female SGE treated groups, (G) different concentrations of female SGE, and (J) SGE and vehicle treated cells for 6 hours. (*P<0.05; **P<0.01).

To detect whether SGE is able to induce apoptosis in peritoneal macrophages, cells were cultured with 20 µg/ml of male and female SGE separately in addition to vehicle controls for 24 hours. Apoptosis was analyzed using Annexin-V/PI double staining for the detection of phosphatodylserine (PS) externalization (an early apoptotic event) and the loss of membrane integrity, which occurs in the late stages of apoptosis. Interestingly, peritoneal macrophages treated with female SGE underwent apoptosis faster than vehicle treated cells; therefore, a significant difference in the percentage of apoptotic cells was observed in both early (P<0.01) and late apoptotic stages (P<0.001). However, the male SGE did not induce apoptosis in either phase (Figure 2 A, B, and C), which may partly account for why the female mosquito transmits infectious diseases while the male mosquito cannot.

Further experiments revealed that, compared with vehicle (14.2%), early apoptosis events gradually increased with increasing concentrations of SGE, such as 24.1% for 5 µg/ml of SGE (P<0.01), 26.6% for 10 µg/ml (P<0.01), and 27.5% for 20 µg/ml (P<0.001). A similar trend was also observed in the late apoptosis phase as illustrated in Figure 2 (A, D, E, F and G).

We next performed a time dependent experiment in which cells were exposed with 20 µg/ml of female SGE at indicated time points. Although insignificant, female SGE induce apoptosis in peritoneal macrophages within 3 hours of incubation. Data obtained at 6 hours showed a significant increase in early (P<0.001) and late apoptosis (P<0.01) in SGE-treated cells compared to vehicle-treated cells (Figure 2H, I and J). The data implied that SGE induce apoptotic events within a few hours, which might be a crucial time to respond against invasion from foreign pathogens.

### Caspase-3 is Involved in *Ar. subalbatus* Saliva- mediated Apoptosis

**Figure 3:**
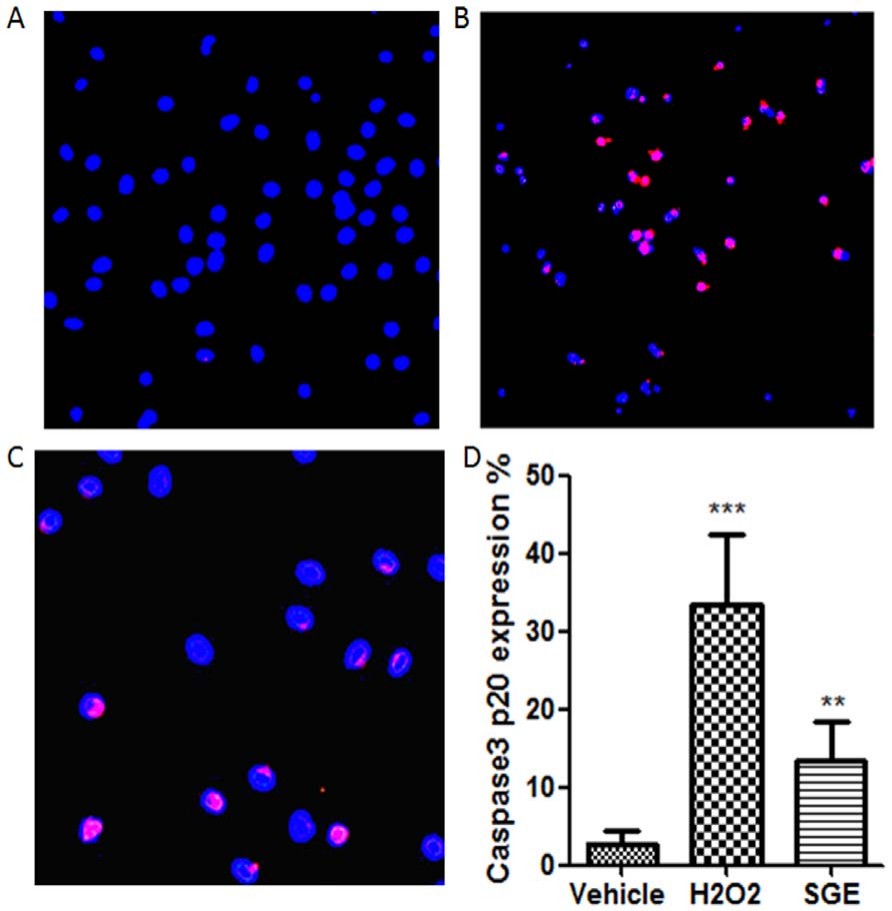
Expression of caspase-3p20 protein in peritoneal macrophages. Peritoneal macrophages were incubated in the presence of (A) vehicle and (B) H_2_O_2_ and (C) female SGE (20 ug/ml) for 24 h. Comparative levels of intracellular caspase-3p20 (red) were observed in female SGE treated cells. DAPI (blue) was used for nucleus staining. (D) The percentages of caspase-3p20 expressing cells were calculated on the basis of the number of positive (red) stained cells in each group. At least 200 cells were counted for each group.

To detect whether *Armigeres subalbatus* SGE-induced apoptosis was caspase3-dependent, cellular expression of caspase-3p20 in SGE or vehicle-treated peritoneal macrophages was analyzed. H_2_O_2_-treated cells served as the positive control. As shown in Figure 3, cellular expression of cleaved caspase-3 was observed in 13.5% SGE-treated cells which was significantly higher compared to that of the vehicle controls (2.75%, P<0.001). However, the expression levels were less than H_2_O_2_ treated cells that served as positive control (Figure 3,A,B,C and D).

### *Ar. subalbatus* Saliva Equally Affects RAW264.7 and THP-1 Cells

**Figure 4:**
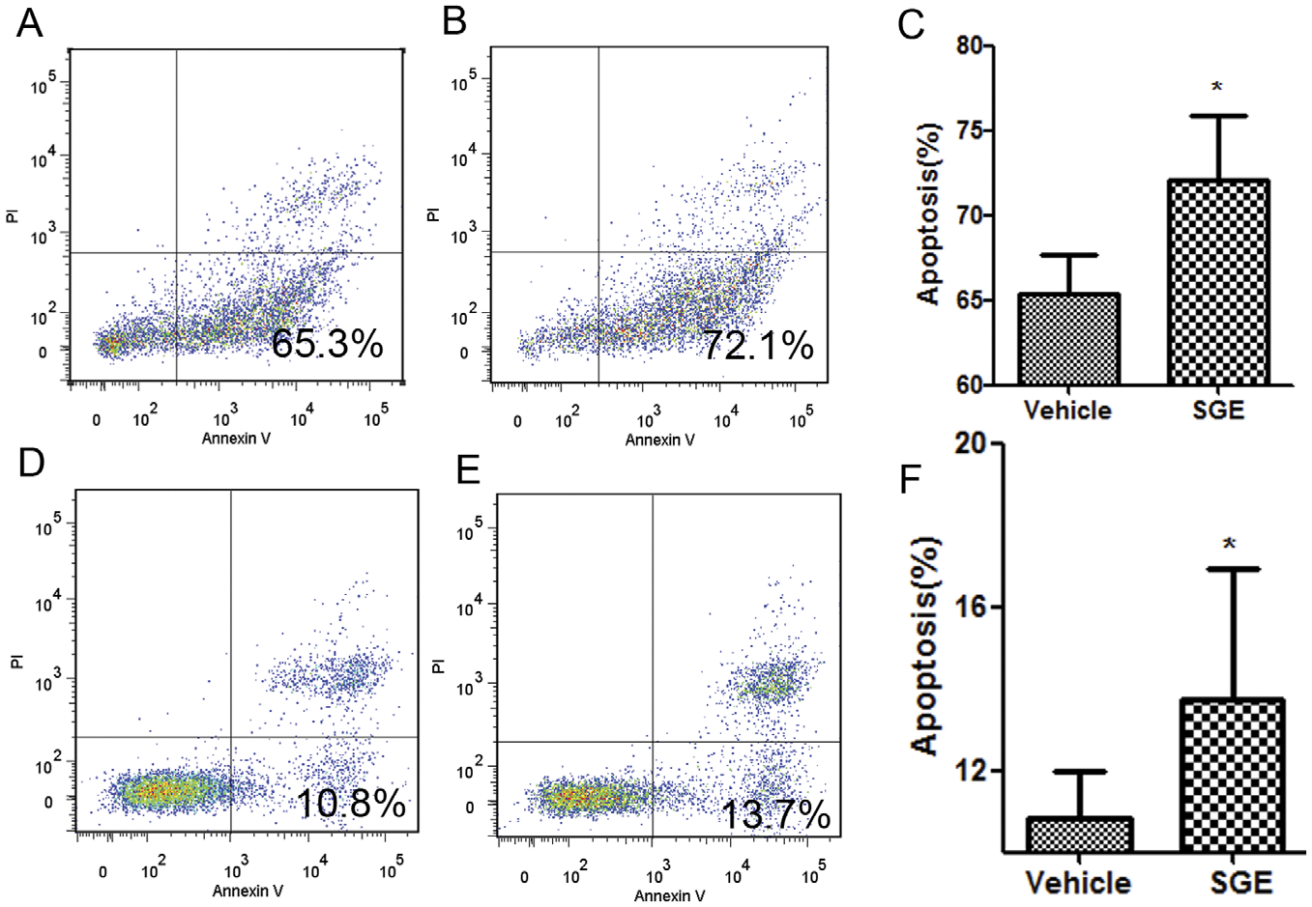
SGE-induced apoptosis in RAW264.7 and THP-1 cells. FACS plots represent apoptosis in vehicle (A) and female SGE (B) treated RAW264.7 cells whereas figures (D) and (E) show the respective treatment of THP-1 cells. Apoptosis was analyzed by Annexin-V/PI staining. AnnexinV-, PI- cells are live cells, Annexin-V+, PI- cells are early apoptotic cells, and Annexin V+, PI+ cells are late apoptotic cells. Bar graphs (C) and (F) represent percentage of apoptotic cells in RAW264.7 and THP-1 cells respectively. Data is presented as mean ± SD. (*P<0.05).

The results from the above-mentioned experiments showing that *Ar. subalbatus* SGE can trigger apoptosis in primary macrophages further stimulated our interest to test whether *Ar. subalbatus* SGE sustain their apoptotic potential against transformed macrophages. We selected two different monocytic macrophage cell lines, RAW 264.7 and THP-1 belonging to murine and human origins respectively. Cells were cultured in the presence or absence of 20 µg/ml of SGE for 24 hours and analyzed by flow cytometry using Annexin V/PI double staining. As shown in Figure 4 early apoptosis was significantly and equally increased in SGE-treated RAW 264.7 (P<0.01) (Figure 4 A, B, C) and THP-1 (P<0.01) cells (Figure 4D, E, F) compared to those incubated with vehicle. This is in agreement with the data acquired with peritoneal macrophages and confirms that apoptosis triggered by *Ar. subalbatus* SGE involve signaling cascade(s) that constitutively present in normal macrophages and is not modified by tumorigenesis.

### *Ar. subalbatus* Saliva-mediated Apoptosis is Independent of Cell-type and Host

**Figure 5:**
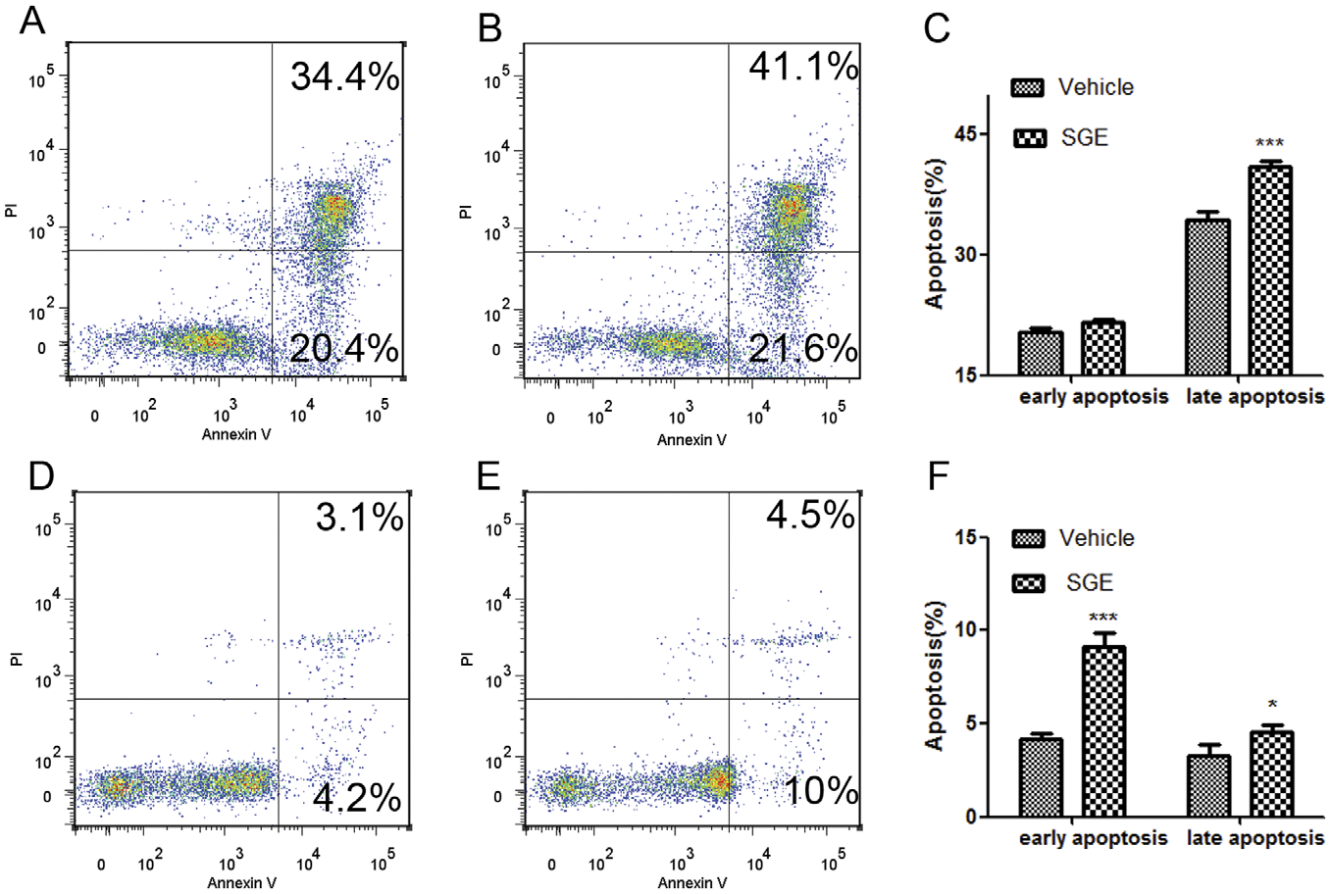
Female SGE can induce apoptosis in different cell types. Splenocytes from BALB/C or human PBMCs were cultured in the presence or absence female SGE (20 ug/ml) for 24 h. Following 24-h incubation, cells were analyzed for apoptosis by Annexin-V FITC and PI staining by flow cytometric analysis. Annexin-V-, PI- cells are live cells, Annexin-V+, PI- cells are early apoptotic cells, and Annexin-V+, PI+ cells are late apoptotic cells. (A) Vehicle and (B) Female SGE 20 ug/ml in splenocytes and (C) the apoptosis levels in splenocytes and (D) Vehicle and (E) Female SGE 20 ug/ml in human PBMCs and (F) The apoptosis levels in the human PBMCs. The dates were expressed as mean ± SD. (*P<0.05; ** P<0.01).

We next examined whether SGE exert similar apoptotic effects in other cell types. For this purpose, lymphocytes isolated from BALB/C spleens were treated with 20 µg/ml of female SGE for 24 hours. Cells were collected for apoptosis, stained with Annexin-V/PI and subjected to flow cytometry. While SGE had little effect on early apoptosis, it was able to significantly induce late apoptosis (P<0.001) (Figure 5 A, B, C). Since host peripheral blood cells are initial responders upon vector biting, we investigated whether SGE exert apoptosis in human PBMCs. Results showed that the apoptosis can be efficiently induced in both the early (P 0.001) and the late phase (P<0.05) in SGE-treated human PBMCs compared to vehicle controls; however, differences were more evident in early apoptosis (Figure 5E, F, G). Since PBMCs are the mixture of mononuclear cells, selective gating strategy was applied to determine if the rate of SGE-induced apoptosis is different among lymphocytes (CD3^+^) and monocytes (CD14^+^); however, no significant differences were observed (data not shown). Collectively, these results helped us to draw the conclusion that *Ar. subalbatus* SGE has the capability to induce apoptosis to all immune responders regardless of their cell type and host.

### *Ar. subalbatus* Saliva Triggers Apoptosis Through Fas Receptor in Caspase-8 Independent Manner

**Figure 6:**
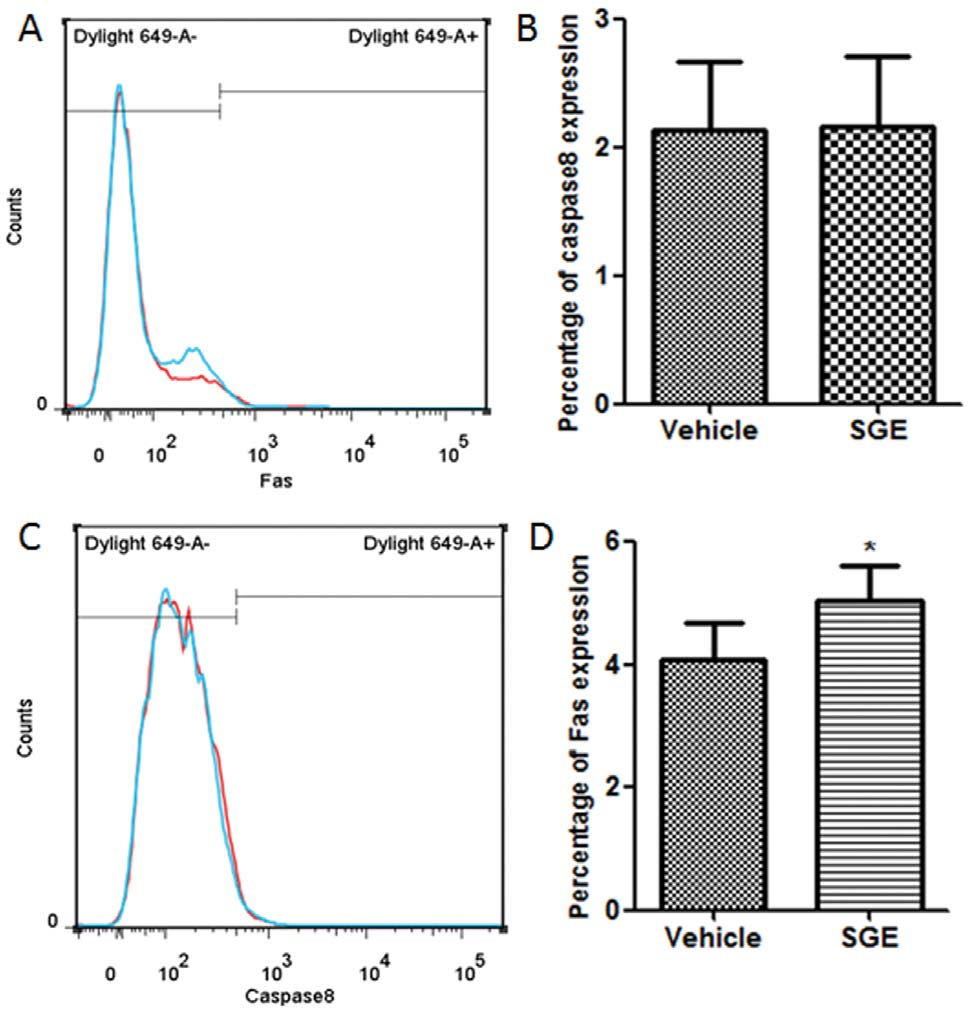
Effect of female SGE on the expression of Fas/CD95 receptor. Peritoneal cells from mice were incubated in the presence or absence female SGE (20 ug/ml) for 24 h.After incubation, cells were incubated with anti-Fas (A and B) or anti-caspase8 (C and D) and secondary antibody labeled with Dylight 649. The analysis was preceded by flow cytometry. The red color represents the Vehicle which are not exposed to female SGE and blue color represents the SGE treated cells. The data were expressed as mean ± SD. (*P<0.05; ).

Since apoptosis was triggered by SGE in a variety of immune cells, we can deduce that components of mosquito saliva can interact with a death receptor which is universally distributed on the surface of immune cells and trigger an extrinsic apoptosis pathway. It is widely known that Fas/CD95 is a death receptor on macrophages and lymphocytes that up regulate upon interaction with exogenous mediators. We measured Fas protein expression on peritoneal macrophages cultured in the presence or absence of SGE for 24 hours. Fas expression was significantly up regulated in SGE-treated cells (5.05%) compared with the vehicle controls (4.09%) P<0.05; however, we did not find up regulation of caspase-8 expression in SGE-treated cells (2.14%) compared to vehicle treated cells (2.14% P>0.05) (Figure 6). The results indicate that *Ar. subalbatus* SGE triggers an extrinsic apoptotic pathway via interaction with the Fas receptor but in a caspase-8 independent manner.

### *Ar. subalbatus* Saliva Mediated Apoptosis Involved p38 Signaling Pathway

**Figure 7:**
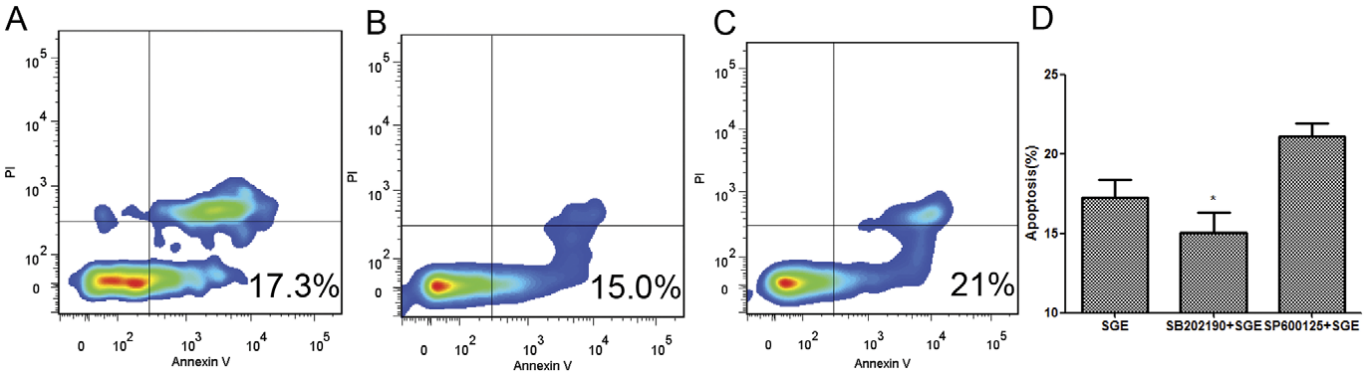
Effect of p38 inhibitor and JNK inhibitor on peritoneal cells from BALB/C and RAW264.7 apoptosis induced by female SGE. Cells were pretreated with or without inhibitor (SB202190 or SP600125, 20 uM/ml) for 30 min. After that, cells were incubated at the presence or absence female SGE (20 ug/ml) for 24 h. Following incubation, cells were analyzed for apoptosis via Annexin-V FITC, PI staining detected by flow cytometry. Annexin-V-, PI- cells are live cells, Annexin-V+, PI- cells are early apoptotic cells, and Annexin-V+, PI+ cells are late apoptotic cells. (A) Female 20 ug/ml SGE and (B) SB202190+female 20 ug/ml SGE and (C) SP600125+female 20 ug/ml SGE. (D) The apoptosis levels in peritoneal cells from BALB/C after p38 inhibitor and JNK inhibitor treatment. The data were expressed as mean ± SD. (*P<0.05).

Fas receptor mediated apoptosis usually involves JNK and p38 MAPKs. To dissect downstream signaling events in the case of *Ar. subalbatus* SGE-induced apoptosis, we used small molecular pathway inhibitors such as SP600125 (JNK inhibitor) and SB202190 (p38 inhibitor) with or without SGE. Data have demonstrated that the indicated concentration of these inhibitors did not have direct toxic effects on peritoneal macrophages (Data not shown). Pretreatment of peritoneal macrophages with SB202190 (p38 inhibitor) was able to significantly inhibit SGE-mediated apoptosis compared those cells which did not receive inhibitor pretreatment (P<0.05) (Figure 7). However, no difference in the percentage of SGE-mediated apoptosis was observed in the presence or absence of SP600125 (JNK inhibitor).

From these observations, we may conclude that *Ar. subalbatus* SGE triggers apoptosis in immune cells by activating p38 downstream signaling pathway.

## Discussion

In vector borne infections the “saliva factor” is a widely discussed issue. A number of studies have previously shown that the addition of arthropod saliva suppresses host inflammatory mediators that eventually lead to the enhancement of pathogen transmission and infection processes [20], [21], [22]. Such interaction is also found in the absence of pathogen, indicating the ability of arthropod saliva to directly interact with immune cells [11]. In previous studies on *Ae. aegypti* mosquito, down regulation of pro-inflammatory mediators such as iNOS, IFN-β and TNF- α together with the increase in anti inflammatory cytokines such as IL-10 and IL4 was observed in primary macrophages which explain the immunomodulatory potential of mosquito saliva [23], [24]. However, the exact mechanism behind this phenomenon is still indefinable. In the present study, we have shown that *Ar. subalbatus* saliva suppresses the expression of pro-infammatory mediators in IFN-γ-induced peritoneal macrophages without changing the expression of IL-10 that rule out the possible Th1/Th2 imbalance. We further explain that *Ar. subalbatus* saliva triggers apoptosis in different immune cells within a few hours; this might be a primary event resulting from a mosquito bite, which in turn inhibits cytokine production and creates a friendly atmosphere for pathogen transmission. In addition, it might generate mixed signals during infection leaving the immune system confused on whether to react first with apoptotic bodies or against the foreign invader.

It is noteworthy that apoptosis is only attributed to female *Ar. subalbatus* saliva and not that of male mosquitoes, which clearly indicates that apoptosis is the saliva of female *Ar. subalbatus* contributes to pathogen transmission. Earlier studies have shown that male and female mosquitoes not only differ in their feeding habits but they also have different transcriptomes [25]. Only blood-feeding female mosquitoes offer a compatible physiological environment to the pathogens that helps in the completion of their life cycle [26], [27]; therefore, it might be possible that the salivary component(s) from female mosquito which are involved in this process, are responsible to trigger apoptosis in host cells.

Apoptosis caused by arthropod saliva is not a new topic in the literature. Previous studies have reported that the saliva of black flies, such as *Simulium vittatum*, as well as that of *Ae. aegypti* cause apoptosis on splenocytes [13], [28], [29]. Since salivary components of blood-sucking arthropods are conserved and may play similar roles in the transmission of diseases [30], sharing protein fingerprints and immune-modulatory kinetics is not unexpected. However, the mechanism of apoptosis induced by arthropod saliva is still indefinable. Unveiling the mechanism, this study for the first time confirms that in macrophages, *Ar. subalbatus* saliva initiates apoptosis upon interaction with the Fas receptor, one of the most prominent death receptors present on the surface of immune cells. Our finding indicates that some salivary components of *Ar. subalbatus* are analogues to FAS molecules; therefore, their proteomic studies would be interesting. Involvement of the Fas receptor also confirms that *Ar. subalbatus* SGE can trigger apoptosis in a wide variety of cells, which was observed in the next set of experiments performed with lymphocytes and human PBMCs. In the context of vector-borne infections, this finding is of particular importance. We already know that to program innate immune response, a mixture of primary responder cells such as macrophages, neutrophils and dendritic cells infiltrate at the site of infection and work together to clear the pathogen which has been transferred through a mosquito bite [31], [32], [33]. One might hypothesize that in these circumstances; SGE-induced apoptosis obstructs primary cells, suppresses effecter mechanisms, and eventually interferes in pathogen clearance. The effect might carry through the adaptive phase since mosquito saliva is able to induce programmed death in lymphocytes; however further studies are necessary to be performed in the presence of pathogens transmitted by *Ar. subalbatus.*

There are two major signaling pathways led by Fas receptor activation in the mammalian system: first through the recruitment of caspase-8 that directly or indirectly induces apoptosis; and second through downstream kinases, such as ASK1, c-Jun NH2-terminal kinase (JNK) and p38 MAPKs [34], that up regulate pro-apoptotic genes either by the activation of specific transcription factors or by directly regulating the activities of mitochondrial pro- and anti-apoptotic proteins [35]. Interestingly, we did not find caspase-8 activation during the process; this observation further stimulated our interest to dissect the exact mechanism. Using a specific inhibitor approach, we treated peritoneal macrophages with JNK (SP600125) and p38 (SB202190) inhibitors for 1 hour prior to the saliva exposure and stained the cells with Annexin-V/PI. Our data showed that SB202190 have the capacity to inhibit the function of saliva, indicating the involvement of p38 MAPK signaling pathway in apoptosis.

Our study has shown that saliva of the female *Ar. subalbatus* is able to induce apoptosis in immune cells via the Fas receptor in a caspase-8 independent manner, but through downstream p38 MAPK signaling. This process initiates within a few hours in key immune cells regardless of their cell type and host and subsequently inhibits cytokines milieu. Given that this process is limited to the female mosquito, which acts as a vector, the data presented here suggest that *Ar. Subalbatus*-induced apoptosis could be functionally important in the context of infectious disease transmission.
